# An increased number of heterozygous calls in the Axiom^TM^ Equine Genotyping Array

**DOI:** 10.1093/g3journal/jkag085

**Published:** 2026-03-31

**Authors:** Annik Imogen Gmel, Ali Pirani, Liz McInnis, Markus Neuditschko

**Affiliations:** Animal GenoPhenomics, Agroscope, Rte de la Tioleyre 4, Posieux 1725, Switzerland; Bioinformatics, Thermo Fisher Scientific, Central Expressway, Santa Clara, CA 95051, United States; Bioinformatics, Thermo Fisher Scientific, Central Expressway, Santa Clara, CA 95051, United States; Animal GenoPhenomics, Agroscope, Rte de la Tioleyre 4, Posieux 1725, Switzerland

**Keywords:** equine genetics, SNP array genotyping, runs of homozygosity (ROH), genotype concordance, batch effects

## Abstract

Single-nucleotide polymorphism (SNP) arrays are commonly used in livestock genetics to investigate complex traits, including genome-wide associations and fine mapping, genomic prediction, and genetic diversity analyses. In a European equine diversity study, we analysed the Axiom Equine 670K SNP genotype data from 2,768 equids representing 20 horse breeds and one donkey breed. Using a strict detection setting to identify genome-wide runs of homozygosity (ROH), 169 purebred horses displayed fewer ROH segments than F1 crosses. Under medium and relaxed settings, ROH counts increased, while some horses continued to exhibit low ROH levels. Therefore, we additionally assessed genotype performance using a four-fold concordance analysis of replicate pairs on the same Axiom batch, between two different Axiom batches, between Illumina EquineSNP50 BeadChip and between Illumina paired-end HiSeq 2000 whole genome sequencing data. Replicates within the same Axiom batch showed the highest average genotype concordance (98.81%), followed by Illumina 50K (97.88%) and whole genome sequencing (96.84%). Re-genotyped horses with few ROH segments showed the lowest concordance (93.52%). According to the SNPolisher classification, 120,838 genome-wide SNPs were not recommended for reproducibility. After calling genotypes of the two different batches together following Axiom Best Practice (e.g. removing failing samples before the final genotyping) and excluding non-recommended SNPs, concordance improved in all comparisons. Therefore, we recommend excluding horses exhibiting an unusually high number of heterozygous calls, using only SNPs with validated genotype performance, and accounting for batch effects when analyzing Axiom Equine 670K SNP genotype data from different batches.

## Introduction

Single-nucleotide polymorphism (SNP) arrays are a powerful genomic technology that enables the simultaneous genotyping of thousands to hundreds of thousands of SNP markers across the genome (Balagué-Dobón et al. 2022). This data resource has become essential in livestock genetics, with key applications including genomic selection (Meuwissen et al. 2001), parentage testing (McClure et al. 2018), disease resistance (Mucha et al. 2015), and genetic diversity studies (Petersen et al. 2013). By enabling the estimation of genomic estimated breeding values (GEBVs), SNP arrays improve selection accuracy and accelerate genetic gains for economically important traits such as milk yield, growth rate, and fertility (Hayes et al. 2009; Weigel et al. 2024). They also support genome-wide association studies (GWAS) and quantitative trait loci (QTL) mapping to identify genes associated with a desirable trait (Signer-Hasler et al. 2012). Additionally, SNP data ensures reliable pedigree verification and can be used to ascertain the genetic diversity within and between breeds, thereby supporting the management decisions to preserve endangered and native breeds (Grilz-Seger et al. 2018). To date, Illumina (www.illumina.com) and Affymetrix (www.thermofisher.com) offer a wide range of customizable and species-specific arrays, including cattle (Boichard et al. 2012; Schopen and Schrooten 2013), pig (Ramos et al. 2009; Ding et al. 2023), sheep (Kijas et al. 2012; Ma et al. 2017), goat (Tosser-Klopp et al. 2014), chicken (Groenen et al. 2011), horse (McCue et al. 2012; Schaefer et al. 2017), and honey bee (Jones et al. 2020). Hence, these two genotype platforms can be considered as key players providing accurate, cost-effective, and high-throughput genotyping services.

SNP array data is now widely used to estimate genomic inbreeding within breeds by identifying genome-wide patterns of runs of homozygosity (ROH), which represent haplotypes that are identical-by-descent (IBD) (Ceballos et al. 2018b). The genomic inbreeding coefficient (F_ROH_) for an animal is calculated by dividing the sum of homozygous segments (S_ROH_) by the total length of the genome. In livestock, previous studies have reported a high concordance between F_ROH_ and pedigree-based inbreeding coefficients (F_PED_) (Purfield et al. 2012). However, in our recent genetic diversity study of Franches-Montagnes (FM) horses, we observed notably lower F_ROH_ values compared to the corresponding F_PED_ values (Gmel et al. 2024a). To further investigate this discrepancy, we re-analysed the Axiom Equine 670K SNP genotype data from a European equine diversity study, which included 2,768 equids across 21 breeds. Furthermore, we performed a four-fold genotype concordance analysis using replicate sample pairs: (1) within the same Axiom batch, (2) between two different Axiom batches, (3) between Axiom and Illumina EquineSNP50 BeadChip, and (4) between Axiom and Illumina paired-end HiSeq 2000 whole genome sequencing data with sequencing depths ranging from 12.69× to 19.63×.

## Material and methods

The Axiom Equine 670K SNP genotype dataset included 2,768 equids, representing 20 horse breeds and one donkey breed, and encompassed both previously published and unpublished data (Table 1).

**Table 1.** Summary of the sampled equids, representing 20 horse breeds and one donkey breed. Breeds are listed in alphabetical order, along with the number of samples (*N*) and the corresponding data source.

No.	Breed	Abbr.	*N*	Data source
*Equus caballus (20 breeds)*				
1	Akhal Theke	AKT	51	Grilz-Seger et al. (2019c)
2	Coldblood Trotter	CT	96	Velie et al. (2020)
3	Einsiedler	ES	54	Gmel et al. (2024b)
4	Exmoor Pony	EXP	278	Velie et al. (2016)
5	Franches-Montagnes	FM	583	Gmel et al. (2024a)
6	French Trotter	FT	156	Grilz-Seger et al. (2019c)
7	Haflinger	HAF	150	Druml et al. (2018)
8	Lipizzaner	LIP	335	Grilz-Seger et al. (2019a)
9	Lusitano	LUS	55	Unpublished data
10	Noriker	NOR	207	Grilz-Seger et al. (2019b)
11	Posavina	POS	30	Grilz-Seger et al. (2018)
12	Pura Raza Español	PRE	15	Unpublished data
13	Purebred Arabian	AR	184	Grilz-Seger et al. (2019c)
14	Selle-Francais	SF	297	Grilz-Seger et al. (2019c)
15	Shetland Pony	SP	4	Grilz-Seger et al. (2020)
16	Shagya Arabian	SA	33	Grilz-Seger et al. (2019c)
17	Slovenian draught horse	SDH	6	Grilz-Seger et al. (2020)
18	Thouroughbred	TB	64	Gmel et al. (2024a)
19	Warmblood	WB	153	Schnider et al. (2017)
20	Zangersheide	ZH	8	Unpublished data
*Equus asinus (1 breed)*				
21	Austrian Hungarian Baroque donkey	AHD	9	Unpublished data

Therefore, detailed information on most of the breeds can be found in the respective original publications. Unlike previous studies, the chromosomal SNP positions were determined based on the EquCab3.0 reference genome (Beeson et al. 2019). For the ROH analysis, we excluded SNPs located on sex chromosomes or those without known chromosomal positions, resulting in a final dataset of 602,131 autosomal SNPs.

Runs of homozygosity segments were identified using an overlapping window approach implemented in Plink v1.9 (Chang et al. 2015), under three parameter settings: strict, medium, and relaxed. The common applied strict setting required a minimum SNP density of one SNP per 50 kb; a maximum gap length of 100 kb; and a minimum ROH length of homozygous segments of 500 kb containing more than 80 homozygous SNPs; up to one heterozygous and one missing SNP were permitted per segment (Grilz-Seger et al. 2019c; Gmel et al. 2024a, 2024b). The medium setting applied the same minimum SNP density (one SNP per 50 kb) and maximum gap length (100 kb) but reduced the minimum ROH length to 300 kb with more than 50 homozygous SNPs; up to one heterozygous and two missing SNPs were allowed per segment. The relaxed setting required a minimum SNP density of one SNP per 50 kb, a maximum gap length of 150 kb, and a minimum ROH length of 300 kb containing more than 30 homozygous SNPs; up to two heterozygous and four missing SNPs were permitted per segment. For each parameter setting and breed, the total number of ROH (N_ROH_), the total length of ROH segments (S_ROH_), and the average length of ROH segments (L_ROH_) were summarized. In addition, F_ROH_ was calculated by dividing S_ROH_ by the length of the autosomal genome (*L*_AUTO_ = 2,280.92 Mb).

Based on the strict and medium ROH results of the horses, we re-genotyped three purebred FM and nine LUS individuals, as these horses exhibited fewer ROH segments compared to F1 outcrosses. Additionally, two FM control horses with an average number of ROH segments were selected for re-genotyping. The genotyping and re-genotyping of the horses was outsourced to Neogen (www.neogen.com) using the same DNA sample. Quality of genotyping was considered acceptable with a dish QC (DQC) ≥ 0.82 and QC call rate (CR) ≥ 97 according to Axiom best practice (Kvale et al. 2015). To further assess genotype concordance within the same Axiom batch, we included 12 technical replicates of the same samples (eight FM and four WB horses). The comparison between two Axiom batches comprised 14 technical replicates (five FM and nine LUS). SNP performance of the 670,806 genome-wide SNPs across both batches was evaluated using SNPolisher classification. Furthermore, we assessed genotype concordance between the Axiom batch and the Illumina EquineSNP50 BeadChip, as well as between the Illumina paired-end HiSeq 2000 whole-genome sequencing data, using technical replicates from 25 and six FM horses, respectively. Therefore, we updated the chromosomal SNP position according to the EquCab3.0 reference genome and determined the number of overlapping SNPs between the Axiom Equine 670K SNP array and the two Illumina platforms containing 46,802 and 476,268 autosomal SNPs, respectively. Genotype concordance was calculated with Plink v1.9 (Chang et al. 2015) using the merge-mode 6 command, which includes only non-missing calls present in both datasets, while no additional filtering (e.g. by call rate or minor allele frequency) was applied prior to the analysis.

## Results and discussion

**Fig. 1.**
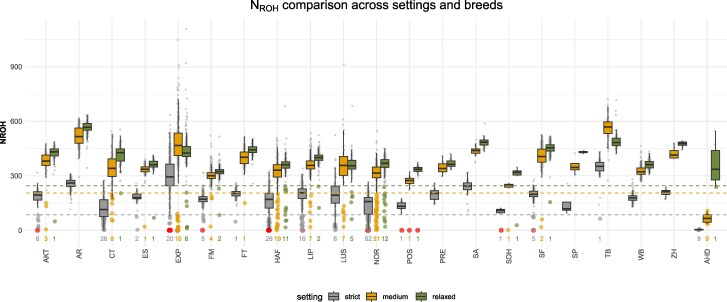
Comparison of the number of runs of homozygosity (N_ROH_) across breeds under three ROH calling settings (strict, medium, and relaxed). Boxplots summarize the distribution of N_ROH_ for each breed and setting. Horizontal dashed line indicates the setting-specific N_ROH_ values observed in F1 outcrosses (strict: N_ROH_ = 85, medium: N_ROH_ = 205, relaxed: N_ROH_ = 245), which were used as thresholds to identify purebred horses with unusually low N_ROH_. Horses with N_ROH_ = 0 are highlighted in red, and individuals with N_ROH_ below the corresponding threshold are emphasized by larger points. Numbers displayed for each breed and setting indicate the number of horses below the threshold, including those with N_ROH_ = 0.

Using the strict ROH setting, we identified 24 horses across nine breeds entirely lacking ROH segments (Fig. 1, red points). Additionally, 145 purebred horses originating from 12 different breeds exhibited fewer ROH segments (N_ROH_ < 85) than observed in F1 WB × FM outcrosses (Fig. 1, grey points). Because F1 individuals are expected to show minimal autozygosity, they provide a biologically meaningful lower bound for ROH. Purebred horses with fewer ROH segments than this threshold might be associated with an elevated number of heterozygous calls due to genotyping artifacts rather than reflecting the true absence of autozygosity. Consequently, only six horse breeds did not include any individuals with an increased number of heterozygous calls under the strict ROH setting. As expected, the number of purebred horses falling below the corresponding F1-derived thresholds decreased under the medium and relaxed ROH settings (N_ROH_ < 205 and N_ROH_ < 245), with 101 and 44 individuals identified, respectively. Nevertheless, three horses were consistently observed to entirely lack ROH segments across all settings. Similar results were observed for S_ROH_, L_ROH,_ and F_ROH_ (Supplementary Figs. 1–3), although F_ROH_ estimates using the relaxed setting were on average 5% higher than previously published results (Gmel et al. 2024a, 2024b). Under the relaxed setting, donkeys also displayed a moderate number of N_ROH_, while they still exhibited the lowest S_ROH_ values across all settings and breeds (Supplementary Fig. 1). This unexpected high heterozygosity in donkeys may reflect a SNP ascertainment bias when calling donkey genotypes alongside horses. However, such biases have been previously linked with an increased number of homozygous calls for breeds that were underrepresented or not included in the SNP discovery panel (Strucken et al. 2021). The performance of the donkey samples may be improved by processing at least 96 samples separately, as recommended in the Axiom Best Practices guidelines. Moreover, it could be noticed that HAF horses associated with a low number of ROH segments also did not carry the respective homozygous allele state for the breed-specific chestnut coat color (Rieder 2009).

**Fig. 2.**
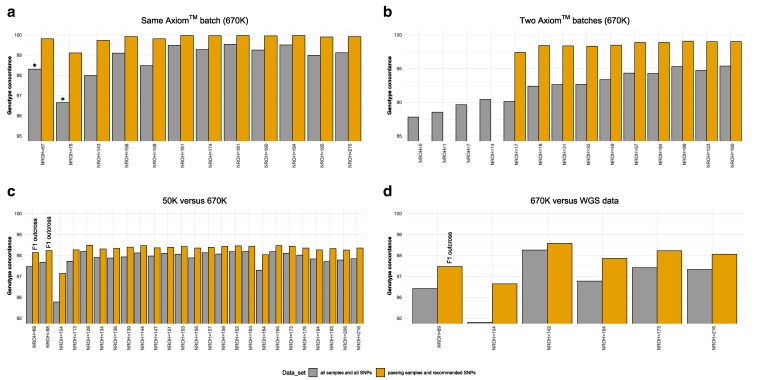
Four-fold genotype concordance analysis. Panels a-d illustrate genotype concordance across four scenarios—same Axiom batch, two Axiom batches, 50K versus 670K, 670K versus whole genome sequencing (WGS)—using two different sample and SNP sets. Each bar represents an individual horse, arranged from left to right by an increasing number of homozygosity segments (N_ROH_) derived using a strict ROH setting. The bar color indicates which sample and SNP set were used for genotype concordance analysis. Replicate pairs within the same Axiom batch exhibiting less N_ROH_ than F1 outcrosses are marked with (*), and F1 outcrosses are also highlighted.

In Fig. 2 (panels A–D, grey bars), we show the N_ROH_ results under the strict setting together with individual genotype concordance rates across the four comparison scenarios. The highest average concordance was observed within replicated pairs processed on the same Axiom batch (98.81%), followed by Illumina 50K array (97.88%), whole genome sequencing (WGS) (96.84%), and comparison across different Axiom batches (93.52%). Two replicated pairs within the same Axiom batch showed lower N_ROH_ values than F1 crosses (Fig. 2, panel A). However, based on the replicate genotype set, these horses carried 109 and 154 N_ROH_ segments, respectively, and exhibited the lowest genotype concordance in that comparison. Notably, the cross-batch comparison included re-genotyped horses with extremely low numbers of ROH segments (N_ROH_  ≤  50), which were associated with reduced concordance rates (Fig. 2, panel B). In contrast, horses with a moderate number of ROH segments (100 ≤ N_ROH_ ≤ 180) demonstrated higher genotype concordance. Additionally, we observed a lower pass rate in the batch containing horses with few ROH segments, suggesting a processing performance issue that contributed to the reduced concordance rates. In the Illumina 50K and WGS comparison, we noticed one horse exhibiting 104 N_ROH_ segments, consistently demonstrating the lowest genotype concordance in both comparisons (Fig. 2, panels C, D). This observation aligns with our previous findings, where this horse also showed low concordance when comparing F_ROH_ against F_PED_ (Gmel et al. 2024a).

Although reduced concordance rates support the interpretation that genotyping artifacts inflate heterozygous calls and thereby reduce the number of ROH segments, alternative factors, including population structure, recent admixture, or structural variation, cannot be entirely excluded. Nevertheless, these influences are unlikely to explain the patterns observed in this dataset. Furthermore, differences in marker density between medium- and high-density SNP arrays and WGS data may influence ROH detection and concordance estimates, which should be taken into account when comparing results across platforms (Ferenčaković et al. 2013; Ceballos et al. 2018a).

During QC of the two merged Axiom batches (192 individuals), we identified four additional failing samples, including a re-genotyped FM horse exhibiting no ROH segments and three LUS horses carrying less than 14 ROH segments, which were therefore excluded from downstream analysis. Following this, we applied the SNPolisher classification to the genome-wide SNP data. The SNPs were allocated into six different categories (Supplementary Table 1). The vast majority (549,968 SNPs) fall into high-quality categories (PolyHighResolution, NoMinorHom, and MonoHighResolution), while a total of 120,838 SNPs were flagged as unreliable and not recommended. These unreliable SNPs were categorized as CallRateBelowThreshold (43,383 SNPs), OffTargetVariant (4,477 SNPs), and Other (72,978 SNPs) and were randomly distributed across the genome without any discernible pattern (Supplementary File 1). After calling genotypes of the two different batches together in accordance with Axiom Best Practice (e.g. removing failing samples before the final genotyping) and excluding non-recommended SNPs, genotype concordance improved in all comparisons (Fig. 2, panels A–D, orange bars): same Axiom batch (99.84%), Illumina 50K (98.33%), WGS (97.81%), and different Axiom batches (98.59%). Therefore, these updated concordance rates are in high agreement with previously reported results comparing Illumina EquineSNP50 BeadChip and Axiom Equine 670K SNP genotypes versus WGS data (Frischknecht et al. 2014; Van Buren et al. 2025).

**Fig. 3.**
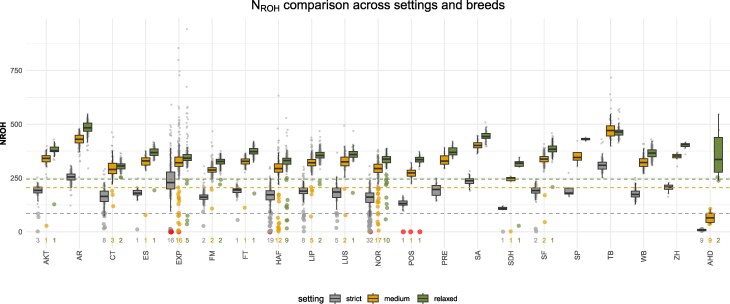
Comparison of the number of runs of homozygosity (N_ROH_) across breeds under three ROH calling settings, using the recommended SNP set. Boxplots summarize the distribution of N_ROH_ for each breed and setting. Horizontal dashed line indicates the setting-specific N_ROH_ values observed in F1 outcrosses (strict: N_ROH_ = 95, medium: N_ROH_ = 210, relaxed: N_ROH_ = 265), which were used as thresholds to identify purebred horses with unusually low N_ROH_. Horses with N_ROH_ = 0 are highlighted in red, and individuals with N_ROH_ below the corresponding threshold are emphasized by larger points. Numbers displayed for each breed and setting indicate the number of horses below the threshold, including those with N_ROH_ = 0.

After excluding non-recommended SNPs, F1 outcrosses consistently showed a higher number of N_ROH_ across all settings (strict: N_ROH_ = 95; medium: N_ROH_ = 210; relaxed: N_ROH_ = 265). As a result, the threshold for identifying purebred horses falling below this level was increased (Fig. 3). Despite the higher threshold, the number of purebred horses with fewer N_ROH_ than F1 decreased across all settings to 99, 64, and 37, respectively. Some breeds, however, still included horses with N_ROH_ equal to zero or very low values (N_ROH_ ≤ 20) across all settings. This is consistent with above observed QC results in FM and LUS horses, which clearly identified these horses as outliers. They exhibited only a slight increase in N_ROH_, and, on average, no significant difference was observed between the two SNP sets. However, for some horses with N_ROH_ just below the F1 threshold, a significant difference between the two SNP sets was detected. Notably, a TB horse exhibited 64 N_ROH_ based on the full SNP set and 251 N_ROH_ under the strict ROH setting on the edited SNP set. In this context, it was also observed that CT, which under the strict ROH setting included 26 purebred horses with fewer N_ROH_ than F1, showed a markedly reduced number (eight) based on the edited SNP set, whereas HAF, with the same number of horses below the F1 threshold, still included 19 purebred horses. Overall, these results suggest that differences in N_ROH_ estimates depend on both the applied ROH settings and the SNP set used, with variability across individual horses and breeds that may reflect differences in genotype performance.

Our results demonstrate that the identification of ROH segments is a reliable indicator for assessing the genotype quality of individual horses, particularly when a low number of ROH segments is observed under strict or medium ROH settings. Accordingly, we recommend excluding horses with an unusually high number of heterozygous calls and limiting downstream analyses to SNPs with validated genotype performance. However, if such low-quality individuals are present in the dataset, genotypes should be re-called after removing these horses to ensure high-quality genotype data. Additionally, we advise accounting for batch effects when analyzing 670K Axiom SNP data from different batches. Given the increased number of heterozygous calls observed in donkeys, we do not recommend calling donkey genotypes alongside horses.

## Supplementary Material

jkag085_Supplementary_Data

## Data Availability

The original (Neogen) and updated (Thermo Fisher Scientific) genotype data for all re-genotyped horses (FM, WB, and LUS) are available in the Supporting Information in standard genotype file formats (Supplementary File 2). Whole-genome sequencing data of FM horses have previously been deposited in the European Variation Archive (EVA) and are available under the project accession number PRJEB28306. Supplemental material available at G3 online.
