# Supplementary material for: An increased number of heterozygous calls in the AxiomTM Equine Genotyping Array
Source: G3 (Bethesda). 2026 Mar 31;16(6):jkag085. doi: 10.1093/g3journal/jkag085 (PMC13232502; doi:10.1093/g3journal/jkag085)
Supplement: jkag085_Supplementary_Data [file jkag085_supplementary_data.zip › Supplementary_Table_1_G3-2026-406574.docx]

**Supplementary Table 1.** SNP classification of the 670,806 genome-wide SNPs

| **SNP category** | **SNPs** | **Percentage** |
| --- | --- | --- |
|  |  |  |
| PolyHighResolution (PHR) | 382,479 | 57% |
| NoMinorHom (NMH) | 119,463 | 17.8% |
| MonoHighResolution (MHR) | 48,026 | 7.2% |
| CallRateBelowThreshold (CRBT) | 43,383 | 6.5% |
| OffTargetVariant (OTV) | 4,477 | 0.7% |
| Other | 72,978 | 10.9% |
| **Total** | **670,806** | **100%** |
