# Supplementary figures and images for: An increased number of heterozygous calls in the AxiomTM Equine Genotyping Array
Source: G3 (Bethesda). 2026 Mar 31;16(6):jkag085. doi: 10.1093/g3journal/jkag085 (PMC13232502; doi:10.1093/g3journal/jkag085)

$S_{ROH}$  comparison across settings and breeds

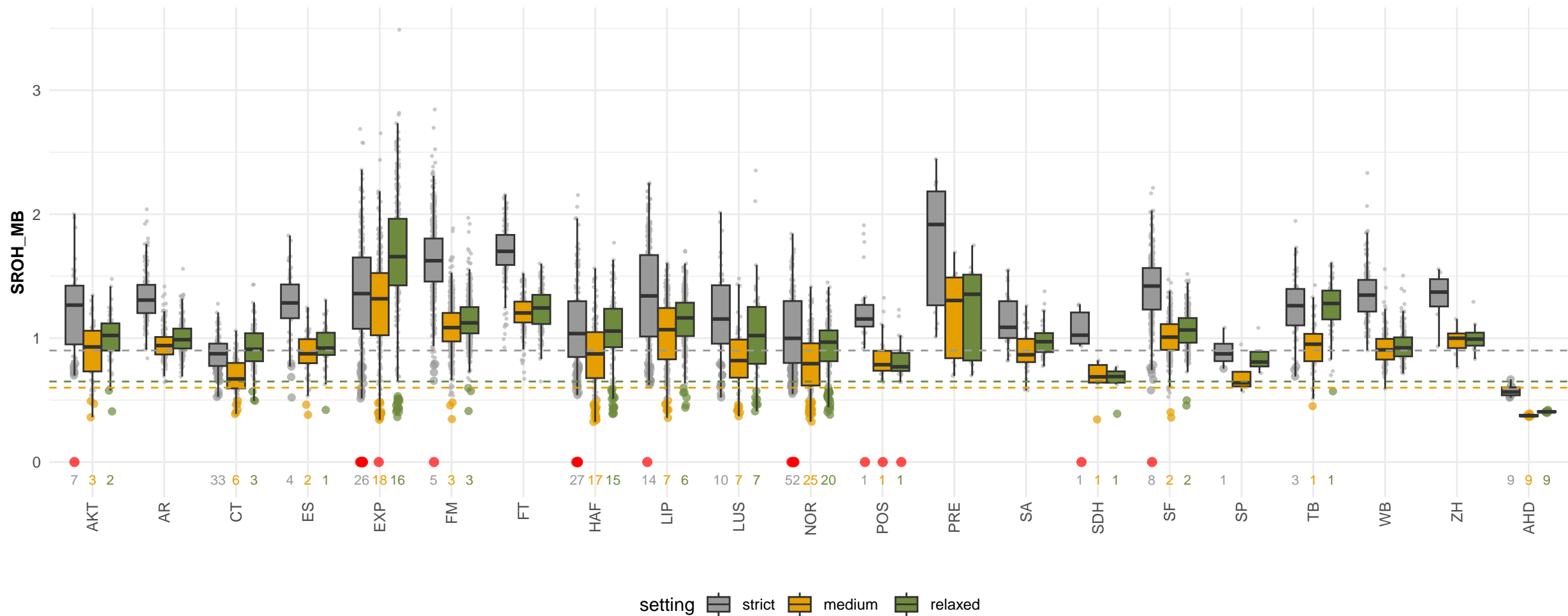

Supplement: jkag085_Supplementary_Data [file jkag085_supplementary_data.zip › Supplementary_Figure_1_G3-2026-406574.pdf]

$L_{ROH}$  comparison across settings and breeds

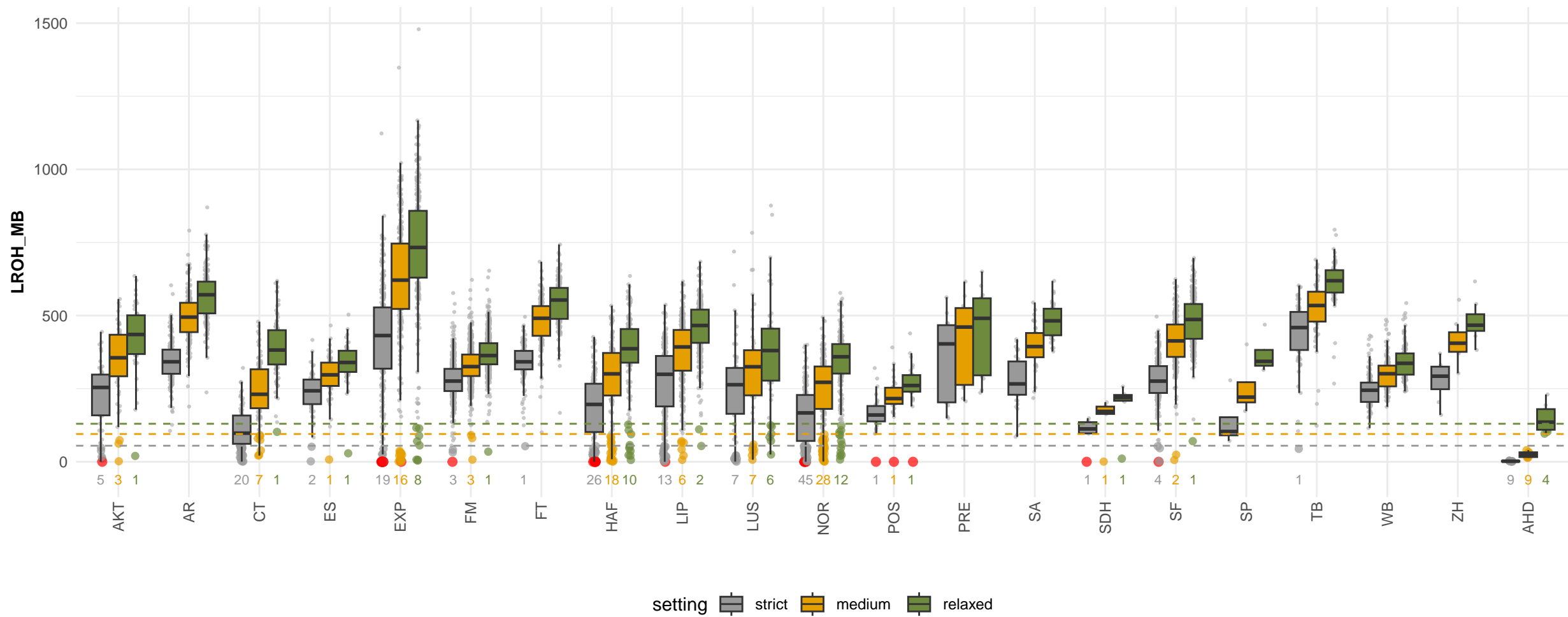

Supplement: jkag085_Supplementary_Data [file jkag085_supplementary_data.zip › Supplementary_Figure_2_G3-2026-406574.pdf]

$F_{ROH}$  comparison across settings and breeds

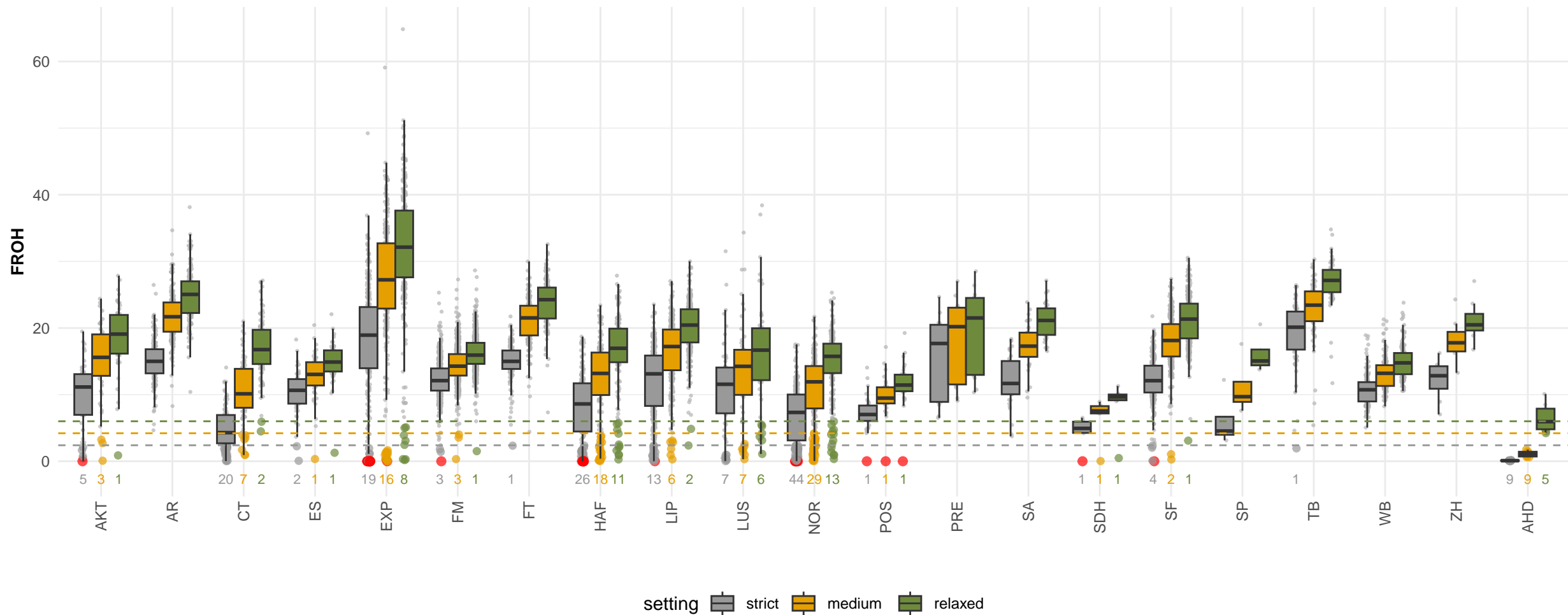

Supplement: jkag085_Supplementary_Data [file jkag085_supplementary_data.zip › Supplementary_Figure_3_G3-2026-406574.pdf]
